# Genomic organization of duplicated short wave-sensitive and long wave-sensitive opsin genes in the green swordtail, *Xiphophorus helleri*

**DOI:** 10.1186/1471-2148-10-87

**Published:** 2010-03-30

**Authors:** Corey T Watson, Krzysztof P Lubieniecki, Ellis Loew, William S Davidson, Felix Breden

**Affiliations:** 1 Department of Biological Sciences, Simon Fraser University, Burnaby, BC, Canada; 2 Department of Molecular Biology and Biochemistry, Simon Fraser University, Burnaby, BC, Canada; 3 Department of Biomedical Sciences, Cornell University, Ithaca, NY, USA

## Abstract

**Background:**

Long wave-sensitive (*LWS*) opsin genes have undergone multiple lineage-specific duplication events throughout the evolution of teleost fishes. *LWS *repertoire expansions in live-bearing fishes (family Poeciliidae) have equipped multiple species in this family with up to four *LWS *genes. Given that color vision, especially attraction to orange male coloration, is important to mate choice within poeciliids, *LWS *opsins have been proposed as candidate genes driving sexual selection in this family. To date the genomic organization of these genes has not been described in the family Poeciliidae, and little is known about the mechanisms regulating the expression of *LWS *opsins in any teleost.

**Results:**

Two BAC clones containing the complete genomic repertoire of *LWS *opsin genes in the green swordtail fish, *Xiphophorus helleri*, were identified and sequenced. Three of the four *LWS *loci identified here were linked in a tandem array downstream of two tightly linked short wave-sensitive 2 (*SWS2*) opsin genes. The fourth *LWS *opsin gene, containing only a single intron, was not linked to the other three and is the product of a retrotransposition event. Genomic and phylogenetic results demonstrate that the *LWS *genes described here share a common evolutionary origin with those previously characterized in other poeciliids. Using qualitative RT-PCR and MSP we showed that each of the *LWS *and *SWS2 *opsins, as well as three other cone opsin genes and a single rod opsin gene, were expressed in the eyes of adult female and male *X. helleri*, contributing to six separate classes of adult retinal cone and rod cells with average λ_max _values of 365 nm, 405 nm, 459 nm, 499 nm, 534 nm and 568 nm. Comparative genomic analysis identified two candidate teleost opsin regulatory regions containing putative CRX binding sites and hormone response elements in upstream sequences of *LWS *gene regions of seven teleost species, including *X. helleri*.

**Conclusions:**

We report the first complete genomic description of *LWS *and *SWS2 *genes in poeciliids. These data will serve as a reference for future work seeking to understand the relationship between *LWS *opsin genomic organization, gene expression, gene family evolution, sexual selection and speciation in this fish family.

## Background

In order to link the fields of evolutionary genetics and behavioral ecology it is critical to understand the influences of genes on behavior [1]. This is especially important for understanding the processes of sexual selection and speciation [2,3]. Opsins are unique among genes known to influence behavior in that it is possible to make explicit mechanistic links between polymorphisms at the amino acid level, wavelength sensitivity at the receptor level, and phenotypic variation at the behavioral level [4]. Opsin genes have recently received a great deal of attention for their roles in mate choice, population divergence and speciation in African cichlids [reviewed in [5]], and have also been posited as candidate genes influencing behavior and sexual selection in the guppy and related species [6-8]. This is not surprising given that variation in opsin genes has facilitated the evolution of color vision across vertebrates [9,10].

Vertebrate opsins make up an intermediate-sized gene family that code for a diverse group of G protein-coupled receptors that initiate light absorbance and phototransduction through their interaction with one of two vitamin-A derived chromophore pigments [9]. The type of chromophore used as well as changes at key amino acid sites in the opsin protein are known to contribute to differences in the wavelength of light that is maximally absorbed (λ_max_) [9]. Cone opsins, which are expressed in cone photoreceptor cells of the retina and responsible for mediating photopic vision, are comprised of four classes able to absorb light at different wavelengths across the spectrum. These are short wave-sensitive opsins (*SWS1*: ultraviolet to blue, and *SWS2*: violet to blue), medium to long wave-sensitive opsins (*MWS *or *LWS*: green to red), and rhodopsin-like opsins (*RH2*: blue to green), all of which were present in the most recent common vertebrate ancestor. The number of functional opsin classes observed in extant vertebrates varies from species to species, contributing to interspecific variation in visual potential [9,10].

In fishes, the evolution of color vision has been directed by opsin gene duplication and diversification, pseudogenization and differential gene expression [11]. This has allowed many species to evolve opsin repertoires and accompanying visual systems that best exploit the photic environments in which they live [12-14]. Furthermore, it is well established in many species that color vision plays a direct role in mate choice and sexual selection [15-18].

Fish in the family Poeciliidae, which includes the guppy and close relatives (*Poecilia *spp.), as well as swordtails and platyfishes (*Xiphophorus *spp.), are long standing models for the study of sexual selection, as both male secondary sexual characteristics and female mate choice are highly variable traits within the group [19-23]. In *Poecilia*, and less so in *Xiphophorus*, male coloration is known to affect female mate choice, although this is perhaps best understood in guppies where females show consistent preferences for males with orange and red color spots [15,24-26]. Furthermore, guppy female preference is known to differ between individuals within and across populations [27-29]. Using microspectrophotometry (MSP), Archer et al. [30] and Archer and Lythgoe [31] provided early mechanistic evidence for variation in guppy visual systems, indentifying expansions in the long wave-sensitive visual capacity of guppies, as well as variation between individuals in the number of long wave visual pigments observed in the retina. Interestingly, molecular data suggest that *Xiphophorus *and *Poecilia *species have expanded their *LWS *opsin repertoires through gene duplication [6-8] corroborating this expansion in long wavelength sensitivity suggested by MSP. Within these poeciliid species, opsin duplication and diversification appears to be highest in *Poecilia *species, which have at least four different *LWS *gene subtypes, compared to that of *Xiphophorus pygmaeus*, the only *Xiphophorus *species so far investigated with regard to *LWS *repertoire, which appears to possess only three [8].

Despite recent molecular work in poeciliids, no complete *LWS *genomic repertoire has been described in any of these species. Obtaining a full description of *LWS *opsins at the genomic level, including genomic organization, total gene copy number and intergenic sequences, will be an important step in elucidating the role that these genes have played in the evolution of this family, whether through gene duplication and divergence, or through differences in gene expression. To date, the genomic organization of cone opsins has been fully characterized in two other teleosts: the zerbrafish, *Danio rerio *[32], and medaka, *Oryzias latipes *[33]. Two *LWS *copies have been identified in each species [32,33]. Genomic data for *LWS *loci are also available in the Ensembl release 56 [34] for *Gasterosteus aculeatus *(stickleback), *Tetraodon nigroviridis *(*Tetraodon*) and *Takifugu rubripes *(fugu), each of which possesses only a single *LWS *gene. Additionally, the *LWS *genes are linked to at least a single *SWS2 *opsin gene in all five of the teleost species mentioned above [32-34]. The genomic organization of *LWS *opsins has also been described in mammals and has contributed a great deal to the understanding of their evolution and function [35-38]. In humans, the characterization of *MWS*/*LWS *opsin gene organization has enabled the identification of regulatory regions essential for the proper expression of these genes and has been vital to the discovery of associations between opsin mutations and human color vision abnormalities [39-42].

In this study we screened bacterial artificial chromosome (BAC) libraries to characterize the genomic organization of *LWS *and *SWS2 *opsins in the green swordtail, *Xiphophorus helleri*, which represents the first complete description of these genes in a member of the family Poeciliidae. We also characterized the spectral sensitivity of adult male and female retinae from this species with MSP and assessed the expression of opsin subtypes by PCR screening of whole eye total RNA. Additionally, using opsin intergenic sequences and cross-species comparisons to other teleosts, we identified potential distal gene regulatory elements including a tentative teleost *LWS *opsin Locus Control Region (LCR), analogous to LCRs previously described for mammalian and other teleost opsin loci.

## Methods

### BAC Library screening

For this study we used the *X. helleri *Bacterial Artificial Chromosome (BAC) library, VMRC27, previously constructed from 12 *X. helleri *males of the Rio Sarabia strain, representing a12-fold genome coverage [43]. Filters and BAC clones from this library were obtained from the Children's Hospital of Oakland Research Institute (CHORI; Oakland, CA, USA). Using *LWS *gene sequences from *Poecilia reticulata *[8], a 60-mer *LWS *opsin specific probe (*lwsprobe*: ACAGCAAAGTTCAAGAAACTTCGTCATCCTCTCAACTGGATCTTGGTCAACCTTGCCATT) was designed from a portion of exon 2 exhibiting high sequence similarity between *LWS *subtypes (100% sequence identity-*S180*, *A180 *and *P180 *subtypes; 91% sequence identity-*S180r *subtype). Both the *LWS *opsin probe and overgo probe (designed to hybridize to filter 'anchor spots' for filter orientation) were end-labelled using ^32^P-ATP and T4 Polynucleotide Kinase (Invitrogen^®^). BAC filter screening was conducted as described by Johnstone *et al*. [44]. Briefly, filter hybridization was carried out overnight at 65°C in 5 × SSC, 5 × Denhardt's solution, and 0.5% SDS. Filters were subsequently washed three times for 60 minutes at 50°C in 1× SSC and 0.1% SDS solution. Hybridized filters were visualized using a Storm 860 phosphoimager (Molecular Dynamics^®^).

### BAC clone characterization and sequencing

The presence of a *LWS *opsin in the BAC clones was confirmed by PCR using *LWS *specific primers (see Additional file 1). BAC shotgun libraries were constructed as described by Johnstone *et al*. [44]. DNA was isolated from BAC clones shown by PCR to possess *LWS *and *SWS2 *loci (VMRC27-80H16, VMRC27-186P13) using a QIAGEN Large-Construct kit. Following isolation the BAC DNA was sonicated, end-repaired, and size selected to 2-5 kb by gel electrophoresis and extracted using a QIAQuick gel purification kit (QIAGEN^®^). Size selected BAC DNA was ligated into Sma I digested, alkaline phosphatase treated pUC19 and used to transform XL1-Blue Supercompetent *E. coli *cells (Stratagene^®^). 1,152 hybrid recombinant clones from the VMRC27-186P13 BAC shotgun library and 1,536 clones from the VMRC27-80H16 BAC shotgun library were sequenced bidirectionally (approximately 1000 bp per clone), providing approximately 7 × and 10 × BAC clone coverage, based on the previously reported average BAC clone insert size of 160 kb [43]. BAC fingerprint contig data [45], and BAC shotgun library sequences were generated at the Michael Smith Genome Sciences Centre (Vancouver, BC, Canada).

### BAC sequence assembly and annotation

Sequences from shotgun libraries were assembled and viewed using the Phred/Phrap and Consed packages [46-48]. The GRASP Annotation Pipeline [49-58] was used for gene annotation (see Additional File 1 for more details). *LWS *nomenclature used for this study was adopted from Ward *et al*. [8]. *LWS *subtypes were differentiated by their "five-site" haplotypes [59], and each named for the amino acid found at the codon position representing the human "180" site. Codon position "180" is one of five key amino acid sites previously shown to contribute to shifts in spectral sensitivity of *MWS*/*LWS *opsin proteins in vertebrates [59]. In the case of the *LWS *subtypes reported here, "S" denotes a Serine, and "P" denotes a Proline at site "180". Names assigned to all other genes, including *SWS2 *opsins, were based on the top BLASTn results and sequence similarity to genes previously reported in other teleost species. SeqManPro (Lasergene 8.0, DNASTAR) was also used for sequence alignments containing BAC assembly consensus sequences and predicted gene sequences.

### RNA extraction and qualitative RT-PCR

One male and one female *X. helleri *from the Rio Sarabia strain obtained from the *Xiphophorus *Genetic Stock Center (San Marcos, TX, USA) were euthanized in NaHCO_3 _buffered Tricaine Methanesulfonate (MS-222). The eyes were removed and total RNA was isolated from male and female specimens separately using the PureLink™ Micro-to-Midi Total RNA Purification kit (Invitrogen^®^). Purified RNA was treated with DNase I as per manufacturer's specifications (Fermentas^®^), and first-strand cDNA synthesis was carried out using 0.2 ug of DNase I-treated total RNA, Oligo dT and SuperScript™ II Reverse Transcriptase (Invitrogen^®^). Using locus specific primers, female and male whole eye cDNA was screened by PCR for the presence or absence of transcripts representing opsin subtypes, as well as the *gephyrin *gene (*GPHN*; explained below). Gene PCR fragments were either sequenced directly after PCR purification or cloned and sequenced (see Additional File 1 for PCR primers). A two round, nested PCR approach was utilized to amplify products for *SWS2A*, *RH2-2 *and *SWS1 *opsins. In the case of *SWS2A *no visible product was produced in the first round, and amplification required a second round of PCR with internal primers. Visible products for both *RH2-2 *and *SWS1 *opsins were produced in first round PCRs, but internal primers were used in a second round to increase locus specificity for direct sequencing of PCR products. In all 2^nd ^round PCR reactions 1:10 dilutions of the 1^st ^round PCR products were used. Cloning of PCR products was carried out using a TOPO TA Cloning^® ^kit with pCR^®^2.1-TOPO^® ^vector and One Shot^® ^chemically competent TOP10 *E. coli *cells (Invitrogen^®^), and purified using a QIAprep^®^Miniprep kit (QIAGEN^®^). PCR products and clones were sequenced at Molecular Cloning Laboratories (MCLAB; San Francisco, CA, USA).

### Microspectrophotometry

MSP was conducted following standard methodology, as described in Loew [60]. Two male and two female *X. helleri *individuals (Rio Sarabia strain) were dark-adapted overnight before being euthanized in NaHCO_3_-buffered MS-222. All procedures were carried out in a darkroom with minimal infrared illumination to prevent bleaching of the photoreceptor cells. Retinas were dissected from the eyes of each fish and placed on a glass slide in a drop of phosphate buffer (pH 7.2 plus 6.0% sucrose) where they were macerated using two razor blades to free the photoreceptor cells and make them accessible for spectrographic measurement. Using a computer-controlled single beam instrument with a 100 W tungsten-halogen lamp, a 40 × mirror objective lens as the condenser, and a 100 × LOMO lens as the objective, individual photoreceptor cell outer segments were scanned from 750 to 350 nm and back at 1.0 nm intervals with odd nm scanned on the downward pass and even nm on the return pass. The selection criteria used for data inclusion into the λ_max _analysis pool were the same as those used by Loew [60]. Each acceptable spectrum was smoothed prior to normalization using a digital filter routine ("smooft")[61]. For curves meeting the selection criteria, the λ_max _(the wavelength at maximum absorbance for a template-derived visual pigment best fitting the experimental data) of the smoothed, normalized (using X_max_) visual pigment absorbance spectrum was obtained using the method of Mansfield as presented by MacNichol [62]. The templates used were those of Lipetz and Cronin [63]. A template curve generated using the calculated λ_max _was overlaid on the raw, unsmoothed data and visually examined for fit.

### Phylogenetic analyses

*LWS *and *SWS2 *opsin coding sequences were obtained from GenBank. *LWS *and *SWS2 *sequences were aligned separately using ClustalW [64] within software package eBioX1.5.1 [65]. Phylogenetic analyses were conducted using PAUP* 4.0 [66]. Trees were reconstructed using Maximum Parsimony (MP) and Neighbor Joining (NJ) methods. Reconstruction of the *LWS *opsin NJ tree employed the General time reversible (GTR) model (with Rate Heterogeneity, alpha = 0.916; Proportion of Invariant Sites = 0.332). Likewise the GTR model (with Rate Heterogeneity, alpha = 0.505) was used to construct the *SWS2 *opsin NJ phylogeny. Best-fit models were chosen using PhyML within the program TOPALi v2 [67]. Missing data were considered using the Pair-wise-Deletion option in the analyses, and node support was calculated using 1000 bootstrap replications for both the MP and NJ trees. MP analyses employed the heuristic near-neighbor-interchange search method. For the *LWS *phylogenies, sequences under the following accession numbers were used: *Poecilia reticulata *EU329431, EU329445, EU329453 and EU329457; *Poecilia bifurca *EU329460, EU329461, EU329465 and EU329466; *Poecilia parae *EU329468, EU329470 and EU329471; *Poecilia picta *EU329473, EU329474, EU329476 and EU329477; *Xiphophorus pygmaeus *EU329478, EU329479 and EU329481; *Oryzias latipes *AB223051 and AB223052; *Gasterosteus aculeatus *BT027981; *Takifugu rubripes *AY598942; *Tetraodon nigroviridis *AY598943; *Danio rerio *AB087803 and AB087804; *Xenopus tropicalis *BC135755; and *Xiphophorus helleri LWS *sequences described in this study. Additionally, for construction of the *SWS2 *trees, we used the following sequences: *Anableps anableps *FJ711152 and FJ711151; *Lucania goodei *AY296737 and AY296736; *Poecilia reticulata *FJ711159 and DQ234860; *Oryzias latipes *AB223056 and AB223057; *Metriaclima zebra *AF247114 and AF317118; *Oreochromis niloticus *AF247120 and AF247116; *Trematomus loennbergii *AY771356; *Cottus gobio *AJ430489; *Gasterosteus aculeatus *BT027452; *Hippoglossus hippoglossus *AF316497; *Pseudopleuronectes americanus *AY631038; *Takifugu rupripes *AY598947; *Tetraodon nigroviridis *AY598948; *Girella punctata *AB158256; *Gadus morhua *AF385822;*Oncorhynchus mykiss *AF425075; *Salmo salar *AY214134; *Danio rerio *AB087809; *Cyprinus carpio *AB113668; *Carassius auratus *L11864; *Xenopus tropicalis *AY177405; as well as the *Xiphophorus helleri SWS2 *sequences characterized here.

### Teleost gene synteny and LCR candidate search

We used web based Genomicus synteny browser [68] and Ensembl genome browsers [34] to assess the synteny between regions sequenced for *X. helleri *in this study and five other teleost genomes for which data were available (Ensembl assembly versions: medaka-HdrR; stickleback-BROAD S1; *Tetraodon*-TETRAODON 8.0; fugu-FUGU 4.0; zebrafish-Zv8). Only genes annotated in these assembly versions were considered for our synteny comparison. Likewise, for the *LWS *regulatory region candidate search, we used available sequence from each of the species listed above, as well as from *Pundamilia pundamilia *[4], a species of African cichlid. Intergenic sequences between *SWS2 *and the closest *LWS *gene from all seven species were analyzed using multipipmaker [69] to locate regions of high sequence conservation (See Additional file 1 for sequences used in the analysis). Identification of potential transcription factor binding sites was done by eye from aligned sequences, using previously identified consensus binding sites for transcription factors with known involvement in opsin gene regulation [70-72].

## Results and Discussion

### *Xiphophorus helleri* opsin genomic organization and gene structure

**Figure 1:**
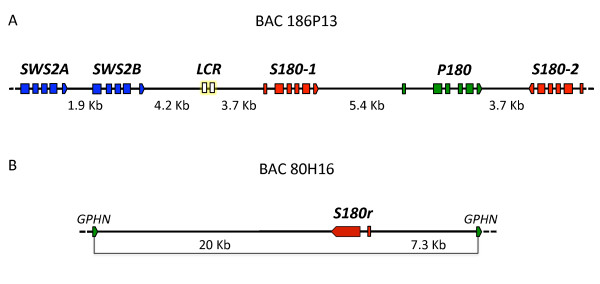
**Genomic organization of *X. helleri LWS*and *SWS2 *opsin genes**. Physical maps of *LWS *and *SWS2 *opsin gene regions sequenced from BAC clones VMRC27-186P13 containing *SWS2A*, *SWS2B*, *S180-1*, *P180 *and *S180-2 *opsin genes (A), and VMRC27-80H16 containing the retrotransposed *S180r *ospin gene and flanking *GPHN *exons (B). Blue, green and red boxes depict the exons of each gene. Final exons of each gene indicate transcriptional direction, and gene names are labelled above each gene. White boxes depict location of Region I and Region II (putative LCR). Approximate intergenic distances are indicated at the bottom of each map between genes.

Using a single 60 bp *LWS *specific probe designed from previously reported *LWS *sequences [6-8] we detected 16 BAC clones positive for *LWS *opsin genes from the *X. helleri *BAC library, VMRC-27 [43]. HindIII fingerprint analysis clustered the 16 BAC clones into two separate fingerprint contigs containing 11 (Contig I) and five clones (Contig II), respectively. The segregation of BAC clones into two separate contigs was expected due to the fact that one of the three previously reported *LWS *genes in *Xiphophorus pygmaeus *is thought to be a retrotransposed gene (*S180r*) [8]. Because retrogenes are usually inserted into regions that are not linked to their ancestral copies [73] we predicted that one of the two fingerprint contigs represented the retrogene locus. To test this hypothesis we used *LWS *locus specific primers (Additional file 1) to screen each of the 16 BAC clones by PCR for the presence of the *LWS *retrogene, *S180r*, and the two other previously reported *LWS *opsins in *Xiphophorus*, *S180 *and *P180 *[8]. All of the Contig II BAC clones tested positive only for the *LWS *retrogene, *S180r*, whereas each of the Contig I BAC clones were positive for either one or both of the other two *LWS *genes but not for *S180r*. Subsequently, because *LWS *genes are linked to *SWS2 *opsins in other teleosts, we tested Contig I BACs for the presence of *SWS2A *and *SWS2B *opsins using consensus primers designed from guppy (DQ234860), *Lucania goodei *(AY296736, AY296737), medaka (AB223056, AB223057), and cichlid (AF247120, AF247116) *SWS2A *and *SWS2B *opsin sequences. A single BAC clone from Contig I (VMRC27-186P13) that was positive for all *SWS2 *and *LWS *opsin PCR primer sets and a single BAC clone from Contig II (VMRC27-80H16) were chosen for shotgun library construction and sequencing, from which we identified complete sequences of four *LWS *(*S180-1*; *S180-2*; *P180*;*S180r*)and two *SWS2 *(*SWS2A*, *SWS2B*) opsin genes (Fig. 1).

BAC clone VMRC27-186P13 (GenBank accession: GQ999832) assembled into two ordered sequence contigs 109 kb and 55 kb in length, and contained a linked gene cluster of three *LWS *(*S180-1*; *S180-2*; *P180*) and two *SWS2 *opsins (*SWS2A*; *SWS2B*; Fig. 1A). The two highly similar *S180 *genes with 99% shared coding sequence identity flank the *P180 *gene on either side. *S180-2 *and *P180 *are positioned on the chromosome in a tail-to-tail orientation, 3,718 bp apart, an organization previously described in *Poecilia *[8]. *S180-1 *is located 5,400 bp upstream of *P180*. The two *SWS2 *copies are tightly linked 1,893 bp apart, and reside 8,563 bp upstream of *S180-1. S180 *and *P180 *subtypes have been described in the genus *Poecilia*, in *X. pygmaeus *[8] and in the anablepid, *Jenysia onca *[74]. It should also be mentioned that sequence in the 3' portion of the *S180γ *subtype in another anablepid, *Anableps anableps*, shares high sequence similarity with *P180 *genes observed in *Poecilia *species [75]. The *A. anableps S180γ *is the likely result of gene conversion [75]. Given that the *P180 *subtype has been identified in *J. onca*, and at least partially in *A. anableps*, the duplication event that produced this locus most likely precedes the split of Poeciliidae and Anablepidae [74,75]. Moreover, the tail-to-tail organization of *X. helleri S180-2 *and *P180 *is identical to that of *S180 *and *P180 *genes described in *P. reticulata*, *P. parae*, *P. picta*, and *P. bifurca *[8]. This provides strong evidence that these genes are orthologs. However, the organization has not been characterized for the *S180 *and *P180 *subtypes described in anablepids [74,75] nor those described in *X. pygamaeus *[8]. It is therefore unclear whether the *S180 *genes described in these species are orthologous to the *X. helleri S180-2 *locus or *S180-1 *locus. The genomic organization of the *SWS2A *and *SWS2B *genes has not been described in any other poeciliid to date; however, *SWS2 *genes are also linked to *LWS *genes in many other species (see below).

The second sequenced BAC clone, VMRC27-80H16 (GenBank accession: GQ999833), assembled into a single 162 kb sequence contig (Fig. 1B) and revealed the presence of a fourth *LWS *opsin, an ortholog of the *S180r *gene previously described in *Poecilia*, *Xiphophorus *and *Lucania *[7,8] and two species from the family Anablepidae [74,75]. It is presumed to be the result of a retrotransposition event as no introns have been observed in PCR products generated from genomic DNA of any of the genera mentioned above [8,74,75]. Until our study only exons II through VI of the *S180r *retroposed gene had been described, and based on the lack of introns it was postulated that this was a completely intronless gene. Here we provide a more complete description of this gene, including a first exon that is homologous to exon I of the other *LWS *genes described here and a single intron. Analyses of human retroduplicates have shown that some retrogenes acquire introns *de novo *following their insertion back into the genome [76-78]. However, in this case the *S180r *exon I and intron I structure and organization are homologous to that observed in *X. helleri S180-1 *and *S180-2*. This homology indicates the *S180r *intron was likely not acquired post duplication, but instead suggests that reverse transcription and genome reinsertion occurred before the first exon of the ancestral *LWS *mRNA transcript had been spliced out. However, the former hypothesis of post duplication intron gain cannot be completely discounted.

This study provides the first examination of the genomic location of the *S180r *gene in relation to other *LWS *gene family members. During the process of retrotransposition, retrogenes can be inserted into regions of the genome unlinked to their ancestral copy and in many cases are inserted into exons or introns of other genes [73]. In line with this trend, we have shown that *S180r *has been inserted into an intron of an unrelated gene, identified here as *gephyrin (GPHN)*. Additionally, because *GPHN *and surrounding genes are not linked to the *LWS*/*SWS2 *gene cluster in other teleosts (see below), it is likely that *S180r *is not linked to the other *X. helleri LWS *genes identified in this study, further supporting the hypothesis that *S180r *is the product of retrotransposition.

**Figure 2:**
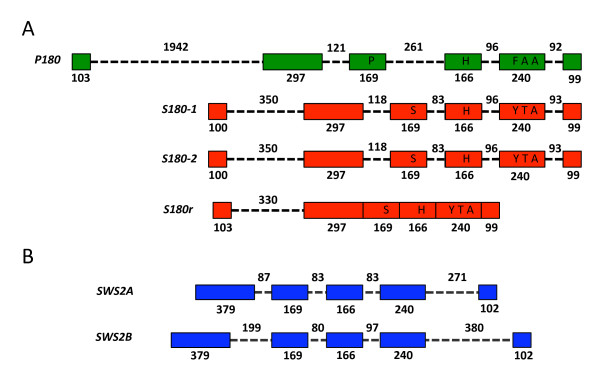
**Exon and intron structure of *X. helleri LWS *and *SWS2 *opsin genes**. Gene exon and intron structures are depicted for each of the *X. helleri LWS *(A) and *SWS2 *(B) opsins described in this study. Exons for each of the genes are represented by colored boxes connected by introns depicted here as dashed black lines. Exon and intron lengths are labelled in base pairs; exon lengths are indicated directly below each exon, and intron lengths are indicated directly above each intron. (A) "five-site" haplotypes are shown for each of the four *X. helleri LWS *genes.

*LWS *opsins *S180-1*, *S180-2*, and *P180 *contain six exons and five introns (Fig. 2A). This feature is shared across vertebrate *MWS*/*LWS *opsins [9]. Fig. 2A shows that exon length is highly conserved across these three *LWS *subtypes. However, introns I and III of *P180 *have undergone increases in size, making them larger than introns I and III of the *S180-1 *and *S180-2 *copies. The *S180r *gene contains only a single intron. The first exon and intron are similar in size to those of the other *LWS S180 *genes, and exon II of *S180r *corresponds to exons II through VI of the other three *LWS *genes (Fig. 2A). Of the four *LWS *opsins, *S180-1 *and *S180-2 *are most similar, differing by only one amino acid caused by a single non-synonymous nucleotide change. However, the genes encoding *S180-1 *and *S180-2 *are different within the 5' and 3' untranslated regions (UTRs). Also shown in Fig. 2A are the "five-site" haplotypes for each *LWS *subtype. The "five-sites" rule has been well established in the vertebrate opsin literature, and refers to five key amino acid sites in the opsin protein that contribute to significant changes in spectral sensitivity [59]. The three *S180 *opsins share the same "five-site" haplotype (SHYTA), and are predicted to absorb light maximally at the same wavelength, whereas the five site haplotype of *P180 *(PHFAA) is different from that observed in the *S180 *subtypes and is expected to have a different λ_max_. It should be noted that *S180r *differs from the other two *S180 *proteins outside the five key sites, sharing only 89% amino acid sequence identity. Weadick and Chang [7] reported amino acid changes specific to the *P. reticulata S180r *protein compared to the other guppy *LWS *subtypes (H247R, A248D, V249I, S256C, K261N, E263Q and R264K), and suggested that this opsin may have increased transducin binding ability, as changes at these sites in bovine rhodopsin-although not the exact changes mentioned above in all instances-are known to be involved in transducin binding [79]. The same changes reported for the guppy *S180r *protein are also observed in the *X. helleri S180r *protein. Additionally, *S180-1 *and *S180-2 *also contain a lysine at amino acid site 264, in place of an arginine, which is found in all non *S180r LWS *opsins reported in the guppy, as well as the *P180 *opsin reported here for *X. helleri*.

*X. helleri SWS2A *and *SWS2B *genes have five exons and four introns (Fig. 2B). A similar structure is found in *SWS2*, *SWS1 *and *RH2 *genes described in other vertebrates [9]. Exon length in the two *SWS2 *genes reported here is identical for exons I through IV, and differs by only 3 bp in exon V. However, as is the case with the LWS opsins described in this study, there are many differences in the length of introns between the two genes, particularly in introns I and IV. All introns of the *LWS *and *SWS2 *opsins reported here contain standard (5' GT-AG 3') splice sites.

### Phylogenetic analysis of LWS and SWS2 sequences

**Figure 3:**
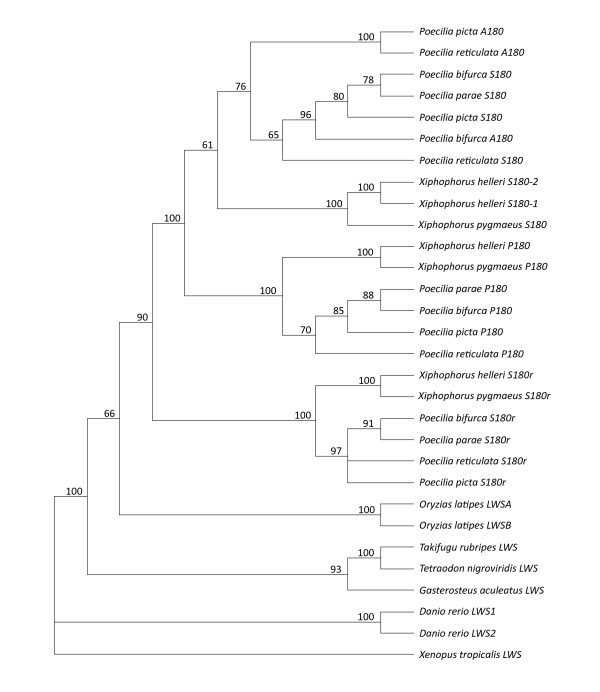
**Neighbor Joining phylogeny of *Xiphophorus *and *Poecilia LWS *opsin genes **. NJ tree was reconstructed using the GTR+I+G model of evolution. Within the tree, values from 1000 bootstrap reiterations are labelled at the nodes. Nodes with values less than 50% were collapsed. *LWS *sequences from the five teleost species used for gene synteny comparisons in this study were also included. The *Xenopus tropicalis LWS *gene sequence was used as the outgroup. Species and gene names are labelled at each of the tips in the phylogeny.

Fig. 3 shows a Neighbor Joining (NJ) tree representing *LWS *opsin evolution in *Xiphophorus *and *Poecilia*. As only partial gene transcripts have been submitted for multiple species and subtypes, sequence lengths used for the analysis range from 621 bp to 1,083 bp. Included in the phylogeny are all *Poecilia *and *Xiphophorus LWS *sequences reported to date, as well as sequences for zebrafish, medaka, stickleback, *Tetraodon *and fugu, because these species were used in synteny analyses (see below). Multiple independent lineage-specific gene duplications are shown in the tree for medaka, zebrafish and within poeciliids. However, it should be noted that the duplication event leading to *S180r *predates the split of Fundulidae, Anablepidae and Poeciliidae [8,74,75]. Additionally, at least two of the other *LWS *genes described in poeciliids and anablepids likely share a common origin [74,75]. *LWS *duplications have also been identified in other teleosts, including the blind cavefish, *Astyanax fasciatus *[80] and *Girella punctata *[81]. Consistent with Ward et al. [8], the poeciliid *LWS *opsins form three clades. Each of the *X. helleri LWS *opsins characterized in this study cluster with sequences from *X. pygmaeus *and *Poecilia *speciesbased on opsin subtype, and one interpretation of the analysis (Fig. 3) is that the *X. helleri S180-1 *and *S180-2 *represent a gene duplication specific to *Xiphophorus*.

It has also been suggested that the *A180 *haplotype, which has so far only been observed in *Poecilia *species, is the product of a genus-specific duplication followed by partial gene conversion with the *P180 *locus [8]. However, as previously discussed by Ward *et al*. [8], the *Poecilia A180 *sequences share high sequence similarity with the *S180 *genes and do not form a single monophyletic group, but rather are interspersed within the clade of *S180 *sequences. Our discovery of a second *S180 *locus in *Xiphophorus *suggests an alternative hypothesis to that of two genus-specific duplications (one in *Poecilia*, producing *A180 *locus, and one in *Xiphophrous*, producing the second *S180*); it is possible that *X. helleri S180-1 *and *Poecilia A180 *are orthologous loci that predate the *Xiphophorus *and *Poecilia *divergence. Under this hypothesis *Poecilia A180 *is the result of gene diversification, as well as possible gene conversion with the *P180 *locus following the divergence of *A180 *and *S180*. Additionally, homogenization of *X. helleri S180-1 *and *S180-2 *genes may have occurred after the divergence of *Poecilia *and *Xiphophorus*. In support of this there is ample evidence for gene conversion from both poeciliids [8] and the closely related anablepids [74,75]. However, data are needed from a wider range of species before the effects of duplication, divergence and gene conversion can be rigorously evaluated.

**Figure 4:**
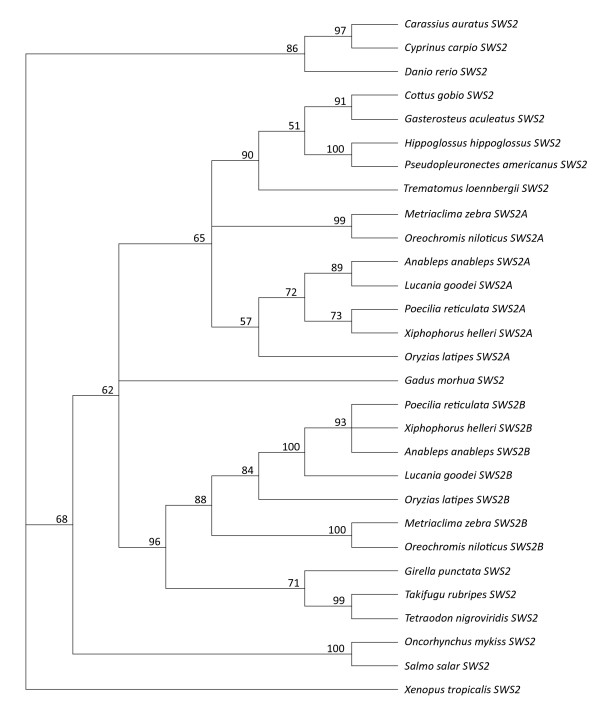
**Neighbor Joining phylogeny of teleost *SWS2 *opsin genes**. NJ tree was reconstructed from *SWS2 *sequences of 20 teleost species and *Xenopus tropicalis *as the outgroup, using the GTR+G model. Within the tree, values from 1000 bootstrap reiterations are labelled at the nodes, and species and gene names are labelled at each of the tips. Nodes with values less than 50% were collapsed.

For the *SWS2 *NJ tree (Fig. 4) we used *SWS2 *coding sequences 412 bp to 1,089 bp in length from 20 species representing a broad range of the teleostei lineage. Unlike the *LWS *gene phylogeny, which is dominated by multiple recent duplication events, the *SWS2 *phylogeny depicts a much older origin for the *X. helleri SWS2 *opsin duplicates at the base of Acanthopterygii. This finding is consistent with previously reported phylogenetic results [33,82,83]. It has also been previously noted that multiple species within Acanthopterygii are represented in the phylogeny by a single *SWS2 *gene (Fig. 4), representing either gene loss or incomplete descriptions of the opsin gene repertoires in those species [33]. Interestingly, species outside of Acanthopterygii with only single genes have evolved *SWS2 *genes to match the spectral sensitivity ranges of either the *SWS2A *or *SWS2B *genes observed in other species [11]. In the case of *X. helleri*, both *SWS2A *and *SWS2B *cluster in the tree with their respective orthologs in *P. reticulata*, *A. anableps *and *L. goodei*. MP trees were also constructed for sequences depicted in the *LWS *and *SWS2 *NJ trees and show only minor differences in topology (see Additional file 2).

### *X. helleri *and teleost synteny

**Figure 5:**
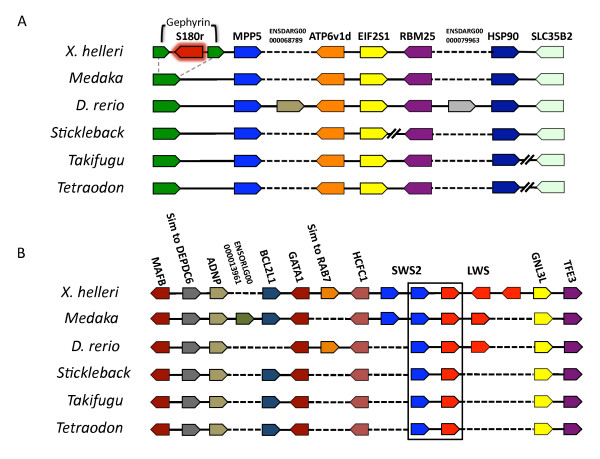
***LWS *opsin genomic regions and teleost gene synteny**. Genomic organization and gene synteny comparisons across teleosts for genes annotated from *X. helleri *BAC clones VMRC27-80H16 (A) and VMRC27-186P13 (B). Picture style was adapted from Genomicus http://www.dyogen.ens.fr/genomicus-57.01/cgi-bin/search.pl. Genes are depicted by colored polygons and transcriptional orientation is indicated at the angled end of each gene as in Fig. 1. Gene names are indicated above the *X. helleri *genes, and orthologs across species are depicted in the same colors. (A) The grey dashed lines from the medaka *GPHN *gene to the *X. helleri GPHN *gene indicate that *S180r *was inserted only within the *X. helleri GPHN *sequence. Double slanted black lines drawn between genes in stickleback, fugu and *Tetraodon *indicate that genes or gene clusters on either side of the black slants are not directly linked in that species. (B) Teleost *SWS2 *and *LWS *opsins are depicted in blue and red. The black box encompassing *SWS2 *and *LWS *opsins across all species represents the organization of these genes in the common vertebrate ancestor. Black dashed lines connecting genes in both (A) and (B) represent the absence of that gene in that particular species.

The results of gene synteny analyses between annotated *X. helleri *BAC sequences and the corresponding regions of five other teleost species are shown in Fig. 5A and 5B. We identified eight complete gene sequences in the BAC VMRC27-80H16, including the *S180r *retrotransposed *LWS *opsin gene (Fig. 5A). *S180r *was not found at this locus in any of the other five teleost genomes used for comparison in this study. The order and orientation of the remaining genes *MPP5*, *ATP6vId*, *EIF2S1*, *RBM25*, *HSP90 *and *SLC35B2 *are conserved in medaka and zebrafish, except that the zebrafish also has two genes (ENSDARG00000068789 and ENSDARG00000079963) not observed in the other species. In stickleback the conserved block of genes mentioned above is disrupted by the translocation of genes *RBM25*, *HSP90 *and *SLC35B2*. A smaller synteny block, including *MPP5*, *ATP6vId*, *EIF2S1*, *RBM25 *and *HSP90*, is also conserved in fugu, and in *Tetraodon*. In both of these species *SLC35B2 *is located at a separate, unlinked locus.

The second region analyzed (Fig. 5B) includes 14 genes annotated from BAC VMRC27-186P13. Most of the variation between species in this region is associated with *LWS *and *SWS2 *opsin gene copy number. Shown in Fig. 5B, *X. helleri *has two *SWS2 *genes and three *LWS *genes. Medaka also has two *SWS2 *copies that are orthologous to the *SWS2A *and *SWS2B *described here in *X. helleri*. Each of the other four species has only a single *SWS2 *copy. Additionally, whereas stickleback, *Tetraodon *and fugu only have a single *LWS *gene, medaka and zebrafish have two. It should be pointed out that these second copies are predicted products of lineage-specific duplication events (Fig. 3). Despite these differences, the synteny block boxed in blue (Fig. 5B) is highly conserved across all of the teleosts analyzed here. This gene organization has also been described in monotremes and most likely represents the ancestral *SWS2*/*LWS *gene organization found in the most recent common ancestor of mammals, fish, birds and reptiles [38,84].

### Retinal cone pigment characterization and opsin gene expression

Assessing the spectral absorbance properties of opsin proteins can be achieved by making comparisons between MSP and molecular opsin sequence data [85] or by visual pigment reconstitution [86]. Moreover, given the extensive amount of effort spent characterizing the effect of amino acid substitutions at key sites in vertebrate *MWS*/*LWS *opsins, broad scale spectral sensitivities can be roughly inferred and assigned to a given opsin protein based on its five site haplotype. This has been attempted for the *LWS *opsins so far identified in the guppy and related species [6-8]. However, in Poeciliidae, no study has yet made direct associations between MSP data and opsin sequences from individuals of the same population and strain.

**Figure 6:**
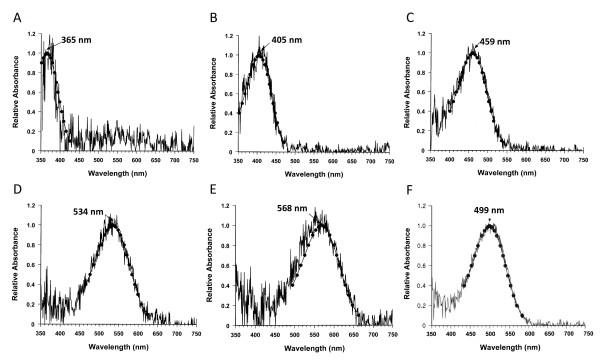
**Adult *X. helleri *MSP data**. Absorbance spectra for pigment classes observed in the retinas of female and male adult *X. helleri*: (A) ultra-violet; (B) violet; (C) blue; (D) green, (E) yellow and (F) rod. Each panel includes template curves generated using the calculated λ_max _overlaid on the raw data. λ_max_values are labelled at the peak of the curve in each panel.

We found that *X. helleri *adult retinas exhibit six separate cone and rod classes defined by differences in maximal spectral sensitivities (Fig. 6). All of the visual pigments fit the typical A1 chromophore pigment-curve and the average λ_max _values observed are as follows; UV cone class (λ_max _= 365 nm; Fig. 6A), violet cone class (λ_max _= 405 nm; Fig. 6B), blue cone class (λ_max _= 459 nm; Fig. 6C), green cone class (λ_max _= 534 nm; Fig. 6D), yellow cone class (λ_max _= 568; Fig. 6E) and a rod class (λ_max _= 499 nm; Fig. 6F). Maximum absorbance averages for the green and yellow cone classes are similar to those described in other poeciliids. The molly, *Poecilia mexicana*, has two *LWS *cone classes with average λ_max _values of 536 nm and 563 nm [87], whereas the guppy retina exhibits one to three with λ_max _values of 533 nm, 548 nm and 572 nm [31]. Although, it should be noted that the 548 nm cone class is proposed to be the result of a mixture of the 533 nm and 577 nm cone classes [31]. Using cDNA synthesized from whole eye total RNA of adult male and female *X. helleri *(Rio Sarabia Strain), products representing each of the *LWS *(*S180-1*, *S180-2*, *P180*, *S180r*) and *SWS2 *(*SWS2B*) opsin subtypes were successfully amplified by PCR, and partial sequences for *RH2-1*, *RH2-2, SWS1 *and *RH1 *opsins were also amplified and sequenced (see Additional File 1 for PCR primers). Partial sequences for *RH2-1*, *RH2-2, SWS1 *and *RH1 *opsins have been deposited under the following GenBank accession numbers (GU454732, GU454733, GU454734, GU454735).

The four *X. helleri LWS *genes described here are grouped into two five-site haplotype classes (Fig. 2A). The *S180 *subtype genes (*S180-1*, *S180-2*, *S180r*) share a common five-site haplotype (SHYTA) and are predicted to exhibit similar spectral sensitivities. This haplotype has also been identified in each of the *LWS *duplicates of both killifish and medaka [33,85]. However, the killifish *LWS *duplicates have a λ_max _at approximately 573 nm, whereas the two *LWS *copies found in medaka exhibit a lower λ_max_, near 560 nm [33,85]. The human *LWS *opsin, which has the SHYTA five site haplotype, also has a λ_max _of 560 nm [59,88,89]. Our MSP data show two peaks in the range predicted to be associated with *LWS *spectral sensitivity, one at 534 nm and another at 568 nm. The lower of these two values, 534 nm, is near the assigned λ_max _of the human *MWS *protein (the human *MWS *arose via duplication of the primate *LWS *locus; ref 34), and one of the cavefish *LWS *proteins [88-90]. Human *MWS *and cavefish *LWS *opsins both share the haplotype AFHAA while the *X. helleri P180 *protein (PFHAA) differs at only a single site. Taken together, these data allow us to conclude that the *X. helleri *peak observed here at 568 nm most likely corresponds to the *S180 *subtypes. However, the fact that we were also able to amplify *RH2 *subtypes from eye cDNA limits our ability to definitively assign the 534 nm cone to the *LWS P180 *gene, as *RH2 *opsins have also been shown to represent cone classes in this spectral range [12,85].

We were unable to amplify *SWS2A *from cDNA using two different sets of primers designed in 5' and 3'UTR sequence, and in exons III and IV (Additional File 1), although a nested PCR approach did result in *SWS2A *amplification. MSP data from this study indicated the presence of a cone pigment class with an average λ_max _of 459 nm (Fig. 6C), which is within the expected range of teleost *SWS2A *pigments [10,12,85,91]. However, *RH2 *opsin absorption spectra as low as 459 nm have also been observed in other teleosts. For example, the *RH2-A *gene in medaka has a λ_max _of 452 nm [33], and the zebrafish *RH2-1 *gene has a λ_max _of 467 nm [32]. Therefore, similar to the 534 nm cone discussed above, it is difficult to conclude which opsin protein is responsible for the 467 nm pigment class. In both of these instances a quantitative PCR approach seeking to ascertain differences in opsin gene expression levels could help clarify these discrepancies.

Compared to *SWS2A *opsins, *SWS2B *genes typically exhibit lower λ_max _values between 405 nm and 425 nm [11]. We detected a violet cone class with a λ_max _of 405 nm, which is the cone pigment class most likely to correspond to the *X. helleri SWS2B *gene. *SWS2B *opsins in both medaka and the killifish have absorption maxima of 405 nm [33,85,91]. Lastly, the fifth cone class described in this study by MSP has a λ_max _of 365 nm, and most likely represents the *SWS1 *gene. Within teleosts, *SWS1 *opsin absorption maxima fall primarily within the ultra violet region of the light spectrum, unlike many other vertebrates, which have evolved *SWS1 *opsins sensitive to longer wavelengths [10,11]. One exception, however, is the scabbardfish *SWS1 *gene, which has evolved a λ_max _of 423 nm [14].

We were also able to amplify PCR products for *GPHN*, suggesting that expression of this gene is not disrupted by the insertion of *S180r*. The insertion of *S180r *into *GPHN *raises interesting questions about the fate of retroduplicates. As mentioned above, retrogenes are typically reinserted at loci unlinked to their ancestral copies. Many examples have recently come to light that suggest retrogenes are able to travel with basic regulatory sequences acquired from their ancestral locus or "hijack" those of their neighboring genes once reinserted back into the genome [73]. It is unknown if the expression of this *LWS *retrogene is facilitated by one of the two mechanisms above or an alternative one. Given that we were able to show expression of the *GPHN *gene, known to be expressed in the zebrafish retina [92], this provides an indication that the chromosomal region where *S180r *now resides is active in the eye of adult *X. helleri*, and may provide a starting point for asking questions about the mechanisms maintaining *S180r *expression. It should be noted that we were unable to amplify *S180r *from cDNA using primers designed within the predicted 5' UTR, suggesting that *S180r *could be a product of gene fusion. Fusion transcripts are sometimes observed in cases in which retrotransposed duplicates have been inserted into other genes [73]. Even though *S180r *and *GPHN *do not have the same transcriptional orientation, we tested for fusion transcripts of the two genes by attempting PCR with two different combinations of *GPHN *and *S180r *primers (Additional File 1), but no products were produced from either of the two combinations used.

### Candidate regulatory elements conserved in SWS2/LWS intergenic sequence: implications for opsin expression

Similar to the *X. helleri LWS *genes described in this study, human *LWS*/*MWS *opsin genes are linked and organized in a tandem array [35]. Retinal specific expression of the human *LWS*/*MWS *genes is regulated by a shared LCR [35-42]. This region has been identified across mammalian taxa [38,39,93], and regions analogous to the mammalian *LWS *LCR have been identified for other opsin gene classes as well [94-96].

**Figure 7:**
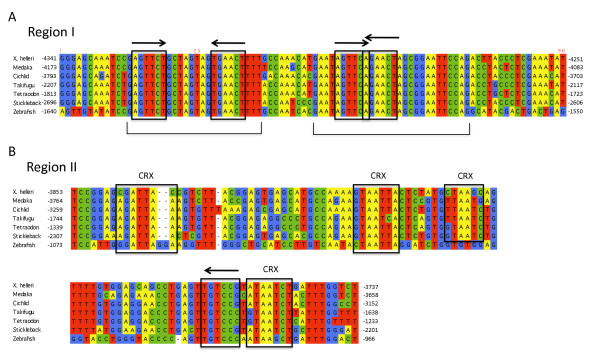
**Potential regulatory regions identified as conserved regions upstream of *LWS *across seven teleost species, including *X. helleri***. (A) Region I: 90 bp of conserved sequence. Black brackets indicate regions with 100 percent shared sequence identity across the seven teleosts shown. Two putative hormone response element (HRE) half-site arrangements are boxed in black. (B) Region II: 106-107 bp of conserved sequence. Four putative CRX binding sites and a single HRE half-site are boxed in black. Black arrows in (A) and (B) indicate sequence direction of HRE half-sites. Bp locations of both regions in relation to the start codon of the *LWS *gene are indicated for each species at the left and right of the alignments.

Using multipipmaker [69] we identified two highly conserved candidate opsin regulatory regions within the intergenic sequence between *SWS2 *and *LWS *opsins in seven teleost species (Fig. 7A, 7B). The two regions, Region I and Region II, are separated by approximately 300-450 bp of sequence (depending on the species) with low phylogenetic conservation. The locations of these regions are indicated in Fig. 7.

Region I, which is located farther upstream of *LWS *opsins than Region II in all species surveyed, is a highly conserved 90 bp stretch containing two conserved blocks of sequence (24 bp long and 29 bp long; Fig. 7A) with 100% shared nucleotide similarity across all seven teleost species. Included within this region are several sequences (Fig. 7A) showing similarity to previously described hormone response element (HRE) half sites [70]. HREs bind nuclear hormone receptors that act as ligand-induced transcription factors following the binding of an appropriate steroid, hormone or vitamin, activating or suppressing gene transcription [97]. Many hormone receptors are known to be involved in the proper development of cone photoreceptor cells [98-101]. In mice, liganded thyroid hormone receptor β2 (TRβ2) is known to activate expression of *MWS *opsins, while both TRβ2 and retinoid X receptor γ (RXRγ) are known to suppress *SWS *opsin expression [98-101]. It has also been shown in juvenile salmonids that thyroid hormone exposure can induce opsin expressional changes in the retina from *SWS1 *subtypes to *SWS2 *subtypes [102]. The preferred HRE half site sequence of TRs and many other non-steroid receptors is based on the sequence PuG [G/T]TCA [70]. However, it should be noted that the sequence of the HRE half site used is dependent on the receptor it is binding, and these sites could be expected to show considerable variation from reported consensus sequences [97]. HRE half-sites typically occur in duplicate and can be arranged as palindromic, direct, inverted or everted repeats [97]. In Region I we identified two candidate HRE palindromic repeat arrangements (Fig. 7A). Half-sites of one of the palindromic repeats are spaced by 8 nucleotides. The other palindromic arrangement contains half-sites with overlapping sequences. In addition to teleosts, we were able to identify Region I upstream of *LWS *opsin genes in the frog, *Xenopus tropicalis*, and lizard, *Anolis carolinensis *(Additional File 3), each of which has physically linked *SWS2 *and *LWS *opsin genes.

The second region identified, Region II, is 106-107 bp in length and compared to region I is only moderately conserved across teleosts. The zebrafish Region II is most divergent from the other species (Fig. 7B), but this is expected considering that the zebrafish is distantly related to the other species analyzed here [103]. Although this region shows weaker conservation across species, it contains a single HRE half-site, similar to those in Region I, and four potential cone-rod-homeobox (CRX) transcription factor binding sites based on previously identified consensus sequences [71,72]. The presence of CRX binding sites is a feature shared with other opsin regulatory regions: the mammalian *LWS *opsin LCR [40-42], the mammalian rod expression region [94], zebrafish *RH2 *opsin LCR [95] and zebrafish *SWS2 *cis-acting regulatory elements [96]. CRX is part of the OTX family of transcription factors and is known to be involved in expression of photoreceptor specific genes [71,72,99]. For example, photoreceptor specific genes, which are partially regulated by CRX, show low retinal expression in mice mutants homozygous for a CRX mutation [104]. Also, deletions involving the human *MWS*/*LWS *LCR, which contains multiple putative CRX binding sites, have been linked to a visual defect known as blue cone monochromacy in which no *MWS *or *LWS *opsins are expressed in the retina [40,41]. The conservation of these non-coding regions across many species alludes to a possible functional role. Wakefield *et al*. [38] postulate that the opsin LCR found in monotremes, which is homologous to that described in other mammals, controls the expression of both the *LWS *and *SWS2 *opsin genes. This could also be true for the putative elements we have uncovered for teleosts. However, further work is needed to assess the role that these regions may play in controlling opsin expression and photoreceptor development, whether separately, or in concert.

Elucidating the function of regulatory elements at this locus in conjunction with what we now know about poeciliid *LWS *opsin organization from this study could prove to be fundamental to our understanding of how *LWS *opsins have influenced sexual selection and speciation in these fish. In the human retina, the expression of *LWS *cones exceeds that of *MWS *cones, and it has been suggested that opsin gene regulatory sequence variation, opsin gene proximity to the LCR, and the three-dimensional chromatin structure of the *LWS*/*MWS *locus may explain these differences [42,105-107]. As in the human *LWS*/*MWS *array, zebrafish *RH2 *opsin gene expression is also dependent on an LCR. It is unknown to what extent the LCR influences spatial and temporal differences in zebrafish *RH2 *expression [95,108], however, proximity to the LCR has been shown experimentally to influence expression levels using artificial expression constructs [95]. Spatial and temporal expression differences have also been observed for zebrafish *LWS *opsins [32,108]. Ward *et al*. [8], using RT-qPCR, showed that the guppy *A180 LWS *gene is expressed at much higher levels compared to the other three *LWS *subtypes. Given that the guppy *A180 *locus is potentially orthologous to the *S180-1 *locus described here for *X. helleri*, it is possible that this gene could also be the closest gene to the LCR in the *Poecilia LWS *array. If this were in fact true, then the proximity of the *A180 *locus in relation to the LCR could be the mechanism driving higher expression levels of the *A180 *gene observed in the guppy. Whether high relative *A180 *expression is a trend across *Poecilia *species remains to be investigated. Indeed, MSP data have shown considerable variation in retinal long wavelength sensitivity within a single guppy population [30,31], suggesting that relative opsin expression levels are not necessarily fixed between individuals of the same population or species. Differences in long wavelength sensitivity have also been found using MSP between species and populations of mollies, which are also in the genus *Poecilia *[87]. However, it is unknown if the differences in visual potential observed in guppies and mollies correspond to variation in female preferences for male coloration patterns [30,31,87]. It will undoubtedly be important to examine to what extent genomic organization, gene copy number variation and opsin promoter sequence polymorphisms affect opsin expressional differences, and in turn how this may contribute to population and species divergence in mate choice in poeciliids, a classic model for the study of evolution by sexual selection.

## Conclusions

We have characterized the genomic organization of four *LWS *and two *SWS2 *opsin genes in the green swordtail fish, *Xiphophorus helleri*. Three of the *LWS *genes (*S180-1*, *S180-2*, *P180*) reside in tandem and are linked to two *SWS2 *opsin genes (*SWS2A*, *SWS2B*), whereas the retrotransposed *S180r LWS *gene is located at a separate unlinked locus. *S180-2*, *P180 *and *S180r *have each been described previously in other species. However, it is unclear whether the *S180-1 *opsin is orthologous to the *Poecilia A180 *gene, one of the three *S180 *genes described in anablepids, or is the result of *a Xiphophorus- or X. helleri*-specific duplication event. Further descriptions of the genomic organization of *LWS *opsin genes in a broader range of species will provide a more definitive understanding of the evolutionary relationships between these genes. Eleven opsins, including the four *LWS *and two *SWS2 *opsin genes described at the genomic level, are expressed in female and male adult retinas, contributing to six retinal cone and rod classes assessed by MSP. To date it is unclear exactly what regulatory mechanisms control the expression of *LWS *and *SWS2 *opsins in *X. helleri *or any teleost, although temporal, spatial and relative expressional differences have been observed in several other species. We have identified two candidate *LWS *opsin regulatory regions. Experiments assessing the function of these regions are currently underway.

## Authors' contributions

CTW carried out molecular wet lab experiments and bioinformatics with assistance and training from KPL and WSD. EL conducted MSP. FB is credited with project design and development, and in conjunction with WSD, was responsible for general project oversight. CTW wrote the manuscript with editing assistance from KPL, EL, WSD and FB. All authors read and approved the final manuscript.

## Supplementary Material

Additional file 1 **PCR primers, and sequences used for Multipipmaker analysis**. Top Left: A list of tools and databases utilized by the GRASP gene annotation pipeline. Top Right: PCR primers used for *LWS *opsin-positive *X. helleri *BAC clones, and for PCR screening of *X. helleri *adult female and male whole eye cDNA. PCR primers from other studies are referenced in "References" column. PCR results and sequencing notes for cDNA PCR screening are also listed in their respective columns. Bottom: Sequences from six teleost species used in addition to those described in this study for *X. helleri *for multipipmaker analyses. For *Pundamilia pundamilia *a GenBank accession number is provided. For all sequences used from one of the Ensembl genome browsers, assembly versions, chromosome/scaffold numbers and base pair positions are listed [100-108]. Click here for file

Additional file 2 ***LWS *and *SWS2 *Maximum Parsimony phylogenies**. MP trees for *LWS *(left) and *SWS2 *(right) genes, constructed from sequences and alignments used for NJ trees in Figs. 3 and 4. Gene sequences for *Xenopus tropicalis SWS2 *and *LWS *genes were used as outgroups. Within the tree, values from 1000 bootstrap reiterations are labelled at the nodes, and species and gene names are labelled at each of the tips. Click here for file

Additional file 3 **Alignment of Region I in *Xenopus tropicalis *and *Anolis carolinensis***. Region I: 90-94 bp of conserved sequence. Black brackets indicate regions with 100 percent shared sequence identity across the species shown. Two putative hormone response element (HRE) half-site arrangements are boxed in black, and black arrows indicate sequence direction of HRE half-sites. Bp locations of both regions in relation to the start codon of the *LWS *gene are indicated at the left and right of the alignments for each species. Click here for file
