# Supplementary material for: Genomic organization of duplicated short wave-sensitive and long wave-sensitive opsin genes in the green swordtail, Xiphophorus helleri
Source: BMC Evol Biol. 2010 Mar 30;10:87. doi: 10.1186/1471-2148-10-87 (PMC3087554; doi:10.1186/1471-2148-10-87)
Supplement: Additional file 1 — PCR primers, and sequences used for Multipipmaker analysis. Top Left: A list of tools and databases utilized by the GRASP gene annotation pipeline. Top Right: PCR primers used for LWS opsin-positive X. helleri BAC clones, and for PCR screening of X. helleri adult female and male whole eye cDNA. PCR primers from other studies are referenced in "References" column. PCR results and sequencing notes for cDNA PCR screening are also listed in their respective columns. Bottom: Sequences from six teleost species used in addition to those described in this study for X. helleri for multipipmaker analyses. For Pundamilia pundamilia a GenBank accession number is provided. For all sequences used from one of the Ensembl genome browsers, assembly versions, chromosome/scaffold numbers and base pair positions are listed [100-108]. [file 1471-2148-10-87-S1.PDF]

Tools and databases included in the GRASP gene annotation pipeline

Alignment Tools.5

BLAST 2.2.17 [50]

BLAT v.32x1 [51]

SSAHA2 1.0.4 [52]

Gene Prediction Tools

Genscan [53]

TMHMM 2.0c [54]

Other

Repeatmasker 3.1.9 [55]

Databases

NCBI non-redundant (nr) Database [56]

Salmo Salar EST Database [49]

TC1 Database

Salmo Repeat Mask Library; repbase_fish.sal_Lib8 [49]

UniRef Database; Release 13.4 [57]

NCBI CDD Conserved Domains Database [58]

Ensembl Organism Genome Protein Database, Release 49 [34]**:**

Danio rerio, Zv7

Gasterosteus aculeatus, BROADS1

Oryzias latipes, MEDAKA1

Takifugu rubripes, FUGU4

Tetraodon nigroviridis, TETRAODON7

Primers used for BAC clone screening

Gene/PCR product	Forward Primer	Reverse Primer	Reference
LWS- S180r	Fw100; GATCCCTTTGAAGGACCAAACT	RevA; CATCCTAGATACTTCCTTCTGGG	Hoffman et al., 2007; Ward et al., 2008
LWS- S180/P180	Fw100; GATCCCTTTGAAGGACCAAACT	Rev5; CATGACTACAACCATCCTGG	Hoffman et al., 2007; Ward et al., 2008
SWS2A	SWS2A-F; ATCCCAGAAGGCCTGCAGTGC	SWS2A.B-R; CGACCACCATCCTGGTCACCT	this study
SWS2B	SWS2B-F; GTCAGCCTTTGGTCTCTTGC	SWS2A.B-R; CGACCACCATCCTGGTCACCT	this study

Primers used for PCR screening of adult *X. helleri* cDNA

Gene/PCR product	Forward Primer	Reverse Primer	Results	Notes
S180r	Xh.S180r-ex1a; CTTGCTCCCTGGAAGAACAAT	RevA; CATCCTAGATACTTCCTTCTGGG	Amplified from Male and Female	Clone and sequenced using T3 and T7 primers
S180r and 5'UTR	Xh.S180r-UTR5; ATCAAGCAGCTCAGATCGTCT	Xh.S180r-R; GGACACTTCAGAGCCGTCAT	Not amplified from either sex	
S180-1	Xh.S180-1-UTR5; CCTCGGGAGGTTACAAGTGA	Xh.S180-1-UTR3; TGCCAGAATGCTATTTAGGTG	Amplified from Male and Female	Clone and sequenced using T3 and T7 primers
S180-2	Xh.S180-2-UTR5; AAGGTTTGACAGCCGAGAGA	Xh.S180-2-UTR3; CTTCATAAACAGAGAACATGAAGG	Amplified from Male and Female	Clone and sequenced using T3 and T7 primers
P180	Xh.P180-UTR5; CCCAGCAAAACTCTCAAGGT	Xh.P180-UTR3; CAGTCCTGGCGCTAATAACAAAC	Amplified from Male and Female	Clone and sequenced using T3 and T7 primers
SWS2A-(1st Round)	Xh.SWS2A-UTR5; GTTTCACGGCTCAGAAAGGA	Xh.SWS2A-UTR3; TTTGCTGTTGTTGGATTGTGA	Not amplified from either sex	
SWS2A- (2nd round)	SWS2A-F; ATCCCAGAAGGCCTGCAGTGC	SWS2-R; CGACCACCATCCTGGTCACCT	Product amplified using these primers in 2nd round of nested PCR	Sequenced directly from purified PCR product using PCR primers
SWS2B	Xh.SWS2B-UTR5; CCATCCCAATGAAGATGAGG	Xh.SWS2B-UTR3; CAGAATGCAATTCTAGAATG	Amplified from Male and Female	Sequenced directly from purified PCR product using PCR primers
RH2-1	RH2-1-F; CACAAGGAAACTAGA-GATGGC	RH2-1-R; CATGATTAAGACACATGGTCCT	Amplified from Male and Female	Sequenced directly from purified PCR product using PCR primers
RH2-2 (1st round)	RH2-2-F; CACAGCCTGAGAAGATGGAG	RH2-2-R; CTGTAATCATTAAGCTGCAGTG	Amplified from Male and Female	
RH2-2 (2nd round)	Xh.RH2-2-F; TATGGGACCCTTAGGCTGTG	Xh.RH2-2-R; GGTAGCTGAGAAGGCAGCTC	Amplified from Male and Female	Sequenced directly from purified PCR product using PCR primers
SWS1 (1st round)	SWS1-F; GGGAACTCAGGGTAAAGATGG	SWS1-R; AACATTATGAAGCTGTGGACAC	Amplified from Male and Female	
SWS1 (2nd round)	Xh.SWS1-F; TTCACCTGCATGCACTCTTC	Xh.SWS1-R; GGTGACAAGCCGGTAGTGTT	Amplified from Male and Female	Sequenced directly from purified PCR product using PCR primers
RH1	RH1-F; AACCACAAGCCGCAACCATG	RH1-R; TGAAGCTTGTGCCTGTTGCTC	Amplified from Male and Female	Sequenced directly from purified PCR product using PCR primers
GPHN	geph-F4; TGGCCTACAAGATAGTGCCAGA	geph-UTR3; TATGTGGACATGCACCGTGA	Amplified from Male and Female	Sequenced directly from purified PCR product using PCR primers
GPHN-S180r fusion 1	geph-R; ATGATTTCAGTGCCCAGGAC	Xh.S180r-R; GGACACTTCAGAGCCGTCAT	Not amplified from either sex	
GPHN-S180r fusion 2	Xh.S180r-ex1-F; GCAGAAGATTGGGGAAAACA	geph-F4; TGGCCTACAAGATAGTGCCAGA	Not amplified from either sex	

Sequences used in Multipipmaker analysis

Species	Chr/Scaff.	GenBank Acc #	Sequence Start	Sequence End	Ensembl Assembly Version
Medaka	Chromosome 5	N/A	27,010,810 bp	27,015,469 bp	HdrR
Pundamilia pundamilia	N/A	AB448597	N/A	N/A	N/A
Fugu	Scaffold_79	N/A	746,180 bp	748,856 bp	FUGU 4.0
Tetraodon	Chromosome 11	N/A	10,120,482 bp	10,122,886 bp	TETRAODON 8.0
Stickleback	groupXVII	N/A	10,619,138 bp	10,627,091 bp	BROAD S1
Zebrafish	Chromosome 11	N/A	25,240,722 bp	25,242,976 bp	Zv8
