# Supplementary figures and images for: Genomic organization of duplicated short wave-sensitive and long wave-sensitive opsin genes in the green swordtail, Xiphophorus helleri
Source: BMC Evol Biol. 2010 Mar 30;10:87. doi: 10.1186/1471-2148-10-87 (PMC3087554; doi:10.1186/1471-2148-10-87)

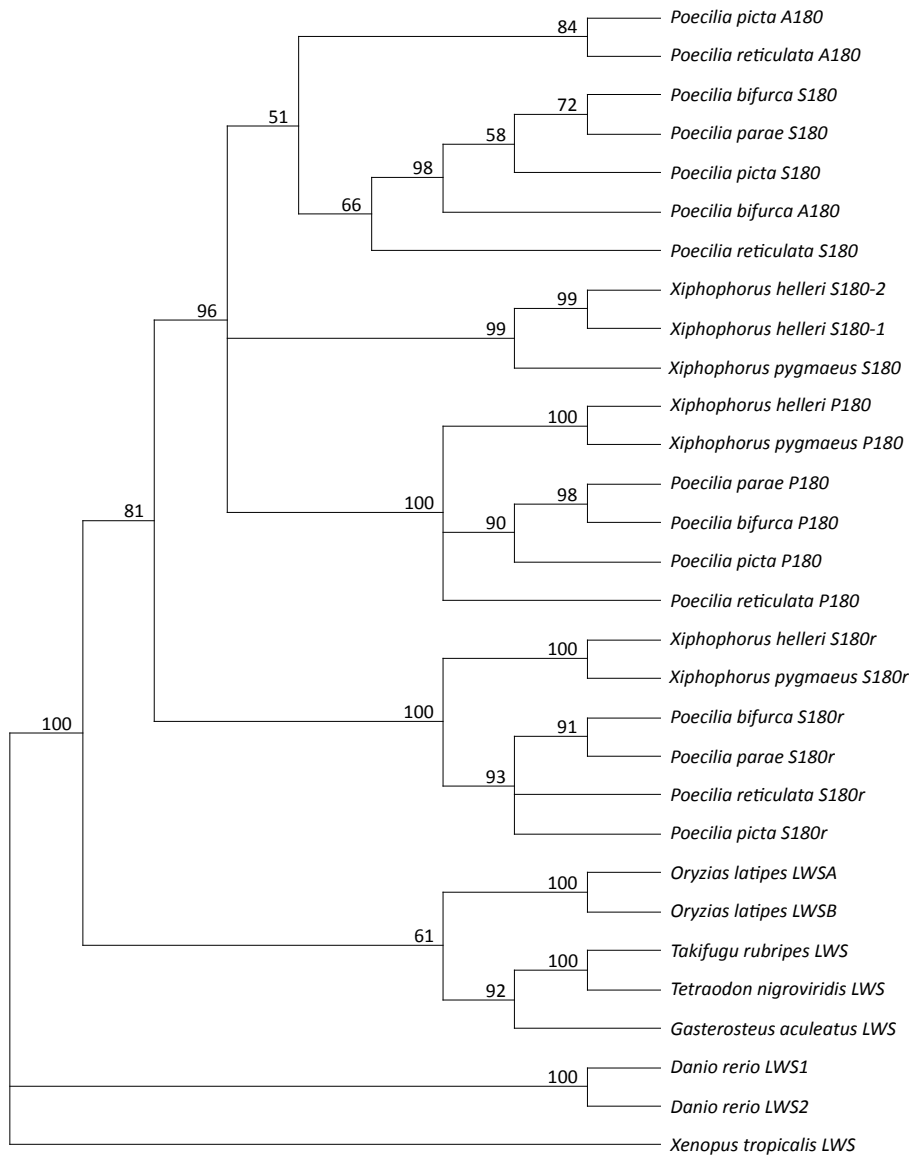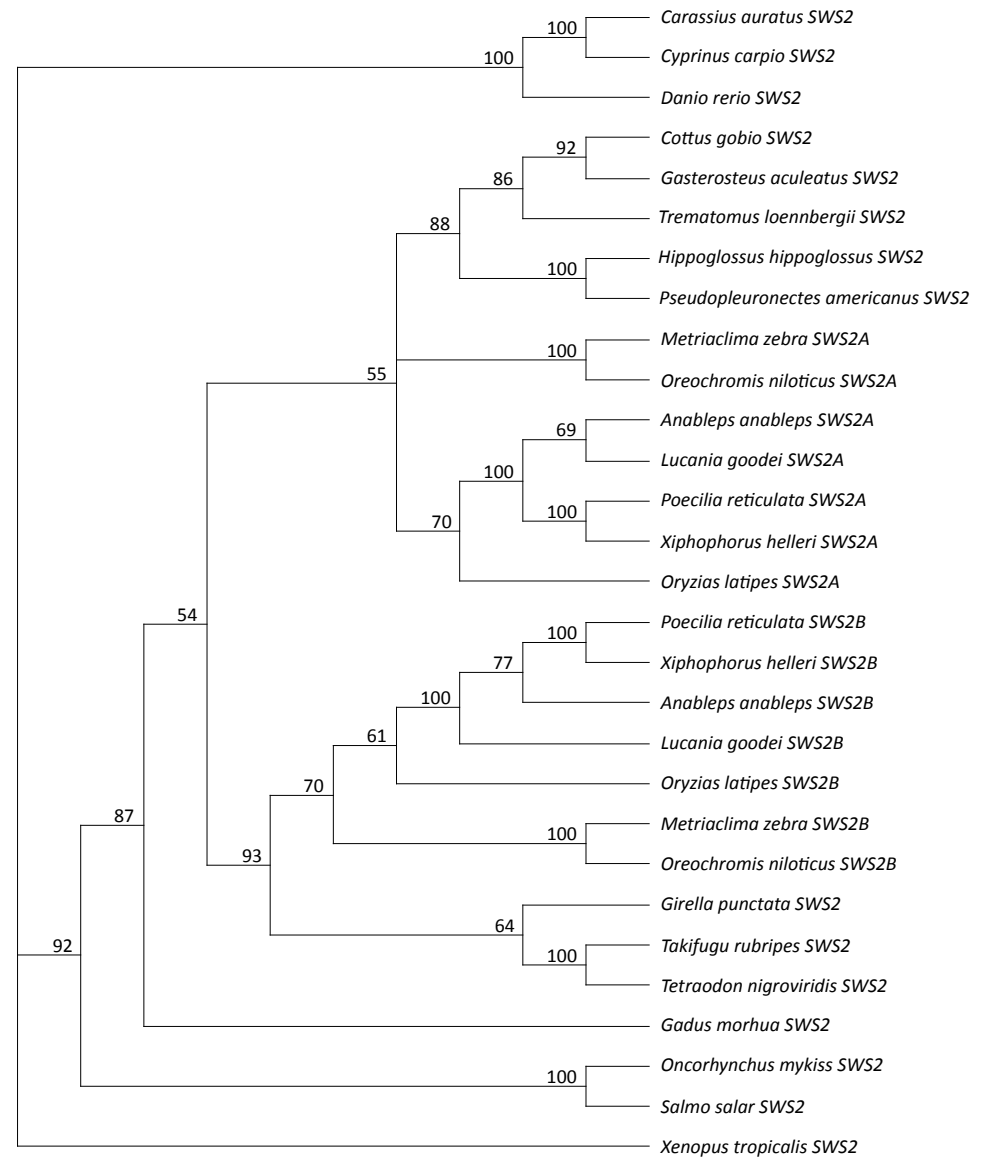

Supplement: Additional file 2 — LWS and SWS2 Maximum Parsimony phylogenies. MP trees for LWS (left) and SWS2 (right) genes, constructed from sequences and alignments used for NJ trees in Figs. 3 and 4. Gene sequences for Xenopus tropicalis SWS2 and LWS genes were used as outgroups. Within the tree, values from 1000 bootstrap reiterations are labelled at the nodes, and species and gene names are labelled at each of the tips. [file 1471-2148-10-87-S2.PDF]

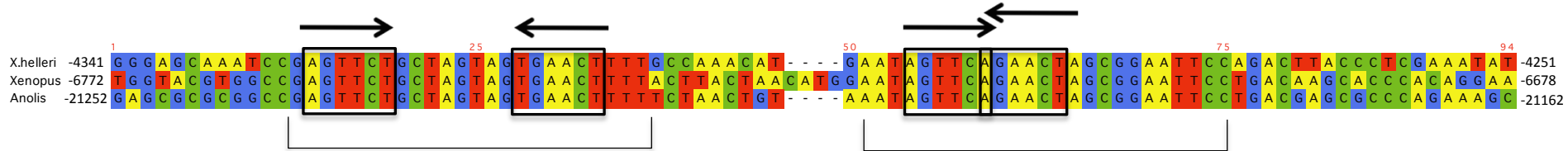

Supplement: Additional file 3 — Alignment of Region I in Xenopus tropicalis and Anolis carolinensis. Region I: 90-94 bp of conserved sequence. Black brackets indicate regions with 100 percent shared sequence identity across the species shown. Two putative hormone response element (HRE) half-site arrangements are boxed in black, and black arrows indicate sequence direction of HRE half-sites. Bp locations of both regions in relation to the start codon of the LWS gene are indicated at the left and right of the alignments for each species. [file 1471-2148-10-87-S3.PDF]
